# RAt-CapsNet: A Deep Learning Network Utilizing Attention and Regional Information for Abnormality Detection in Wireless Capsule Endoscopy

**DOI:** 10.1109/JTEHM.2022.3198819

**Published:** 2022-08-16

**Authors:** Md. Jahin Alam, Rifat Bin Rashid, Shaikh Anowarul Fattah, Mohammad Saquib

**Affiliations:** Department of Electrical and Electronic EngineeringBangladesh University of Engineering and Technology 61750 Dhaka 1000 Bangladesh; Department of Electrical EngineeringThe University of Texas at Dallas 12335 Richardson TX 75080 USA

**Keywords:** Wireless capsule endoscopy, deep CNN, GI tract, attention mechanism, pyramid

## Abstract

**Background**: The emergence of wireless capsule endoscopy (WCE) has presented a viable non-invasive mean of identifying gastrointestinal diseases in the field of clinical gastroenterology. However, to overcome its extended time of manual inspection, a computer aided automatic detection system is getting vast popularity. In this case, major challenges are low resolution and lack of regional context in images extracted from WCE videos. **Methods**: For tackling these challenges, in this paper a convolution neural network (CNN) based architecture, namely RAt-CapsNet, is proposed that reliably employs regional information and attention mechanism to classify abnormalities from WCE video data. The proposed RAt-CapsNet consists of two major pipelines: Compression Pipeline and Regional Correlative Pipeline. In the compression pipeline, an encoder module is designed using a Volumetric Attention Mechanism which provides 3D enhancement to feature maps using spatial domain condensation as well as channel-wise filtering for preserving relevant structural information of images. On the other hand, the regional correlative pipeline consists of Pyramid Feature Extractor which operates on image driven feature vectors to generalize and propagate local relationships of pixels from WCE abnormalities with respect to the normal healthy surrounding. The feature vectors generated by the pipelines are then accumulated to formulate a classification standpoint. **Results**: Promising computational accuracy of mean 98.51% in binary class and over 95.65% in multi-class are obtained through extensive experimentation on a highly unbalanced public dataset with over 47 thousand labelled. **Conclusion**: This outcome in turn supports the efficacy of the proposed methodology as a noteworthy WCE abnormality detection as well as diagnostic system.

## I. Introduction

Failure in early detection of gastrointestinal (GI) tract diseases may cause severe effects and can become cancerous and even can lead to death [1]. Investigating GI tract abnormalities using medical imaging technologies is, however, very challenging. Endoscopy and colonoscopy are two commonly used processes for examining the GI tract, but the procedures are very painful and they cannot cover the entire GI tract. As a painless and efficient alternative, wireless capsule endoscopy (WCE) [2] is getting vast popularity where a capsule camera travels all through the GI tract and generates a large number of images, where tentative abnormality location may constitute a very small percentage of these images. Considering the tedious process of manual observation of these WCE images and high cost of capsule, development of an abnormality automatic detection scheme has recently received great attention amongst the enthusiasts; machine learning and deep learning, to name a few. Researchers aiming towards biomedical innovation are using computerized automatic techniques to further develop diagnosis systems. Research requires fast hardware and cloud-based computers are recently being used as free and user-friendly tools in this case. Publicly available server-based computation power is being procured in the cloud to remotely process, diagnose and enhance biomedical images such as, WCE without thinking about the hardware constraint with great efficiency; effectively carrying out significant research. Limited server access time, however, is a small limitation of cloud computers; unless paid for premium versions with unlimited access period and faster hardware.

## II. Related Works

It is found that in many automatic GI disease detection scheme from WCE images, different machine learning algorithms are applied to perform binary classification between normal and a single disease class; from diseases namely bleeding, ulcer, erosion and angiectasia. For instance, bleeding detection is widely attempted using different methodologies, such as color histogram-based features [3], block-based histogram feature extraction [4], cluster based statistical feature extraction [5], inter-plane intensity variation profile [6] as well as pixels of interest (POI) evaluation by a linear separation scheme [7]. Similarly, for ulcer detection in [8], locally computed features are extracted from HSV colour space representation. Aside from single disease detections, a multi-class scheme is utilized in [9] where a Least-Square Saliency Transformation (LSST) is proposed to identify the salient POI of WCE images.

Recently, deep learning techniques are gaining much attention for automatic GI disease detection from WCE images because of its capability of providing very satisfactory performance without depending on various hand crafted features. However, considering the availability of large dataset, most of the deep learning based GI disease detection methods deal with endoscopy and colonoscopy images [10], [11], [12]. Use of efficient deep learning techniques for classifying GI diseases from wireless capsule endoscopy images faces tremendous challenges due to severe data scarcity. As a result, most of the deep learning oriented methods for WCE image analysis resort to very simple CNN architectures. For example, in [13], two popular CNN architectures, namely AlexNet [14] and GoogLeNet [15] are utilized for successfully classifying between ulcer and normal classes. However, availability of less number of training and testing WCE images may cause biased estimate. Furthermore, in [16], normal and ulcer images are classified using the Xception [17] CNN. In [18], a CNN based semantic segmentation approach is proposed to correctly detect angioectasia disease. In another study for angioectasia detection [19] a CNN based single shot multibox detector is used. For the detection of GI bleeding using deep learning, in [20], an 8-layer CNN is proposed. In a later work [21], they showed that for a limited dataset, an integration of CNN and handcrafted feature extraction method can perform better than their previous work [20]. Aoki *et al.* [22] trained a CNN network on 27,847 images for blood detection and achieved an accuracy of 99.89%. Also, a total comparative analysis among the classification methods of endoscopy images presented in Medico GI challenges over the years 2017, 2018, and 2019 is presented in [23].

Branching away from single disease detection with respect to normal images, classifying two or more GI diseases from given WCE videos is also an important task. It is to be noted, such classification is widely addressed in endoscopy and colonoscopy datasets; however rarely addressed for the case of WCE videos because of the severe scarcity of annotated WCE data. In [24], erosions and ulceration disease detection is carried out from WCE videos collected from 115 patients and it utilizes a CNN based single shot multibox detector. Classification of ulcer and erosion is also attempted by another research group in [25] where they use AlexNet [14] and WCE images are collected from 144 patients. However, it is a matter of fact that development of any innovative deep learning based scheme to simultaneously handle binary as well as the multi-class GI disease classification using WCE images under accessible datasets in particular is rarely attempted and still remains to be a challenge to overcome.

In this paper, RAt-CapsNet, a deep CNN based lightweight network is proposed to classify binary as well as multi-class GI diseases from WCE images. This network is composed of two major pipelines: Compression Pipeline and Regional Correlative Pipeline. In the compression pipeline, Volumetric Attention Mechanism (VAM) enhances image characteristics related to abnormalities in wireless capsule endoscopic images. Moreover, in the regional correlative pipeline, Regional information aggregating Pyramid Feature Extractor (PFE) extracts local relationships of pathological findings with respect to the neighbourhood. Since the proposed network for abnormality detection from capsule endoscopy employs regional information as well as attention mapping mechahnism, it is termed as ‘RAt-CapsNet’. Rigorous experimentation is carried out on a publicly available WCE dataset to demonstrate the performance of the proposed RAt-CapsNet in classifying gastrointestinal diseases and abnormalities.

## III. Dataset Description

The utilized dataset for this study is the Kvasir Capsule [26], which is a large open access wireless capsule endoscopy dataset. This dataset consists of a total 117 videos with 14 classes of findings collected from examinations undertaken at a Norwegian Hospital. In the original examination, the videos were captured in a resolution of 336 $\times $ 336 and the frame rate was 2 frames per second. A total of 4,741,504 image frames were extracted from these videos. After being analyzed and verified by selected medical personnel, 47,238 frames were labelled around findings from 14 different classes including both landmarks and abnormalities. The 3 landmark classes (Ileocecal Valve, Pylorus, Ampulla of Vater), Foreign Bodies and Reduced Views are not linked to any pathological aberrations.

Additionally, some classes have too little samples (Blood-Hetain, Polyp, Erythema) which will hardly assist the training process. Therefore, the subset of classes taken under consideration and introduced to the methodology are: normal, ulcer, erosion, blood, angiectasia and lymphangiectasia. The unbalanced nature of the selected classes found in the dataset is shown in Table 1.

**TABLE 1:** WCE Image Distribution in the Dataset

Class Types	Images	Class Types	Images
Normal Clean	34,339	Blood	446
Ulcer	854	Lymphangiectasia	592
Erosion	506	Angiectasia	866

## IV. Methodology

This section illustrates the steps undertaken in order to distinguish amongst images with significant attributes; attributes which are crucial for medicinal decision-making. A novel CNN network is proposed which is trained on the images under the selected classes to pinpoint the attributes or abnormalities of any given image after performing augmentation-based pre-processing.

### A. Augmentation Oriented Upsampling

**Fig. 1.**
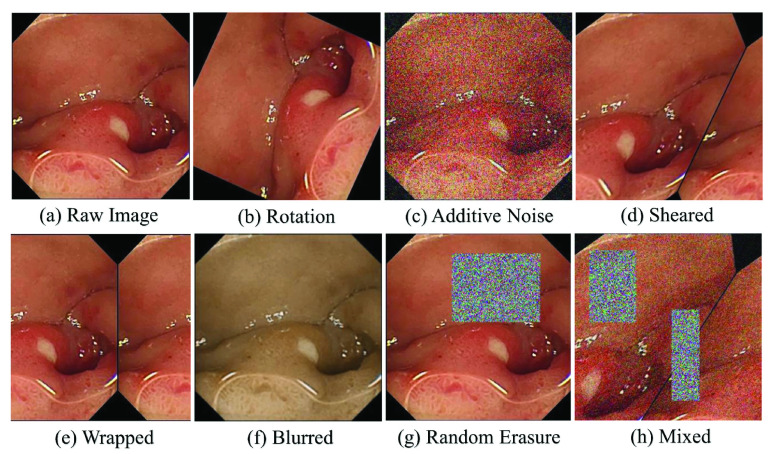
Different types of augmentation techniques.

WCE images are not well distributed among each class. Because of the unbalanced dataset, image augmentation allows the model to get acquainted to a large variety of images in the training phase. Among various types of augmentation techniques, it is found that positional augmentation (i.e: Random Rotation, Flipping, Shearing, Wrapping) and colour augmentation (i.e: Random erasing, Random additive noise, Image blurring) can overcome the effects of class imbalance. In Fig. 1, different types of implemented augmentations are depicted.

### B. Proposed Network

This sub-section illustrates the proposed network scheme which is intended to extract characteristic features to enable classification for wireless capsule endoscopy images. There are two distinct challenges, namely (i) low image resolution, (ii) scarcity of contextual information. The WCE data structure and properties are dissimilar to endoscopy and colonoscopy images as they do not have high resolution details compared to the latter two instances. Therefore, isolating and detecting an abnormality that may or may not reside on the gastrointestinal mucosa is a challenging task. As for the second challenge, the mucosal wall may be exposed to too little or too much light and this may create anomalies during detection of a pathological aberration. In that case, distinguishing the required attributes from the surrounding area becomes another challenge. A necessity arises to capture precisely the regional information surrounding the abnormality irrespective of pixel intensity. The proposed scheme is designed in such a way as to address both challenges.

**Fig. 2.**
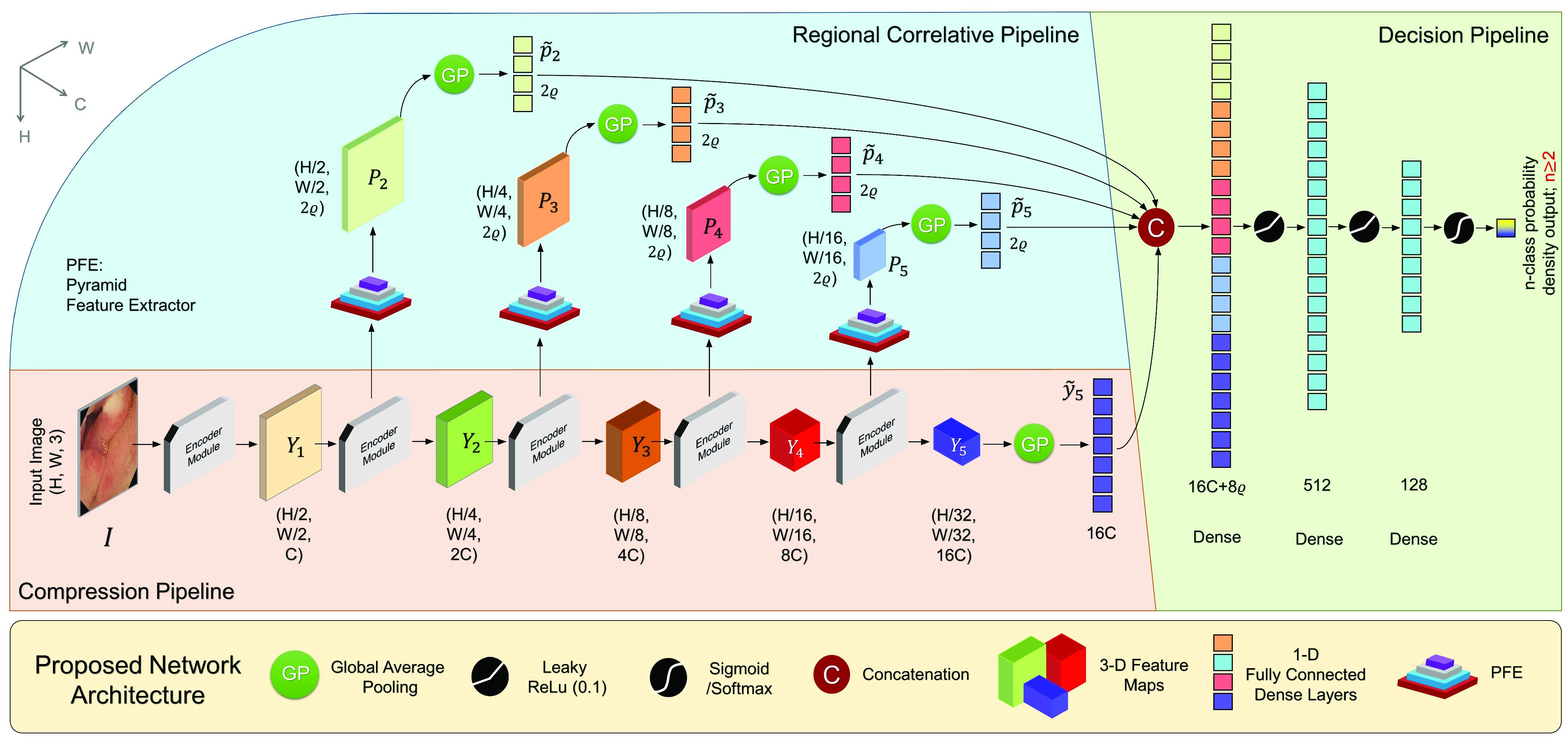
Proposed RAt-CapsNet architecture: It comprises of a Compression Pipeline for dimensional reduction and base feature propagation; a regional correlative pipeline for estimating region concentrated feature aggregation and extraction; and a decision pipeline in order to attain viable probability densities with regards to image attributes.

**Fig. 3.**
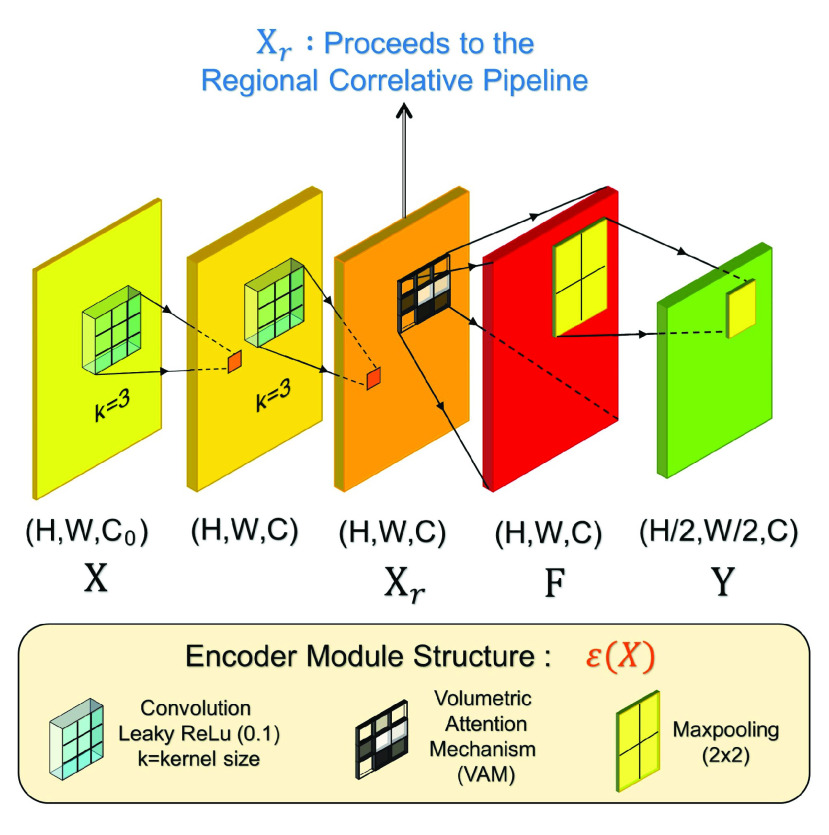
Proposed encoder module utilized within the Compression Pipeline, represented as $\varepsilon (X)$. It takes a feature map $X$ and produces a dimensionally reduced output $Y$. Additionally, the registered $X_{r}$ is sent to the regional correlative pipeline.

**Fig. 4.**
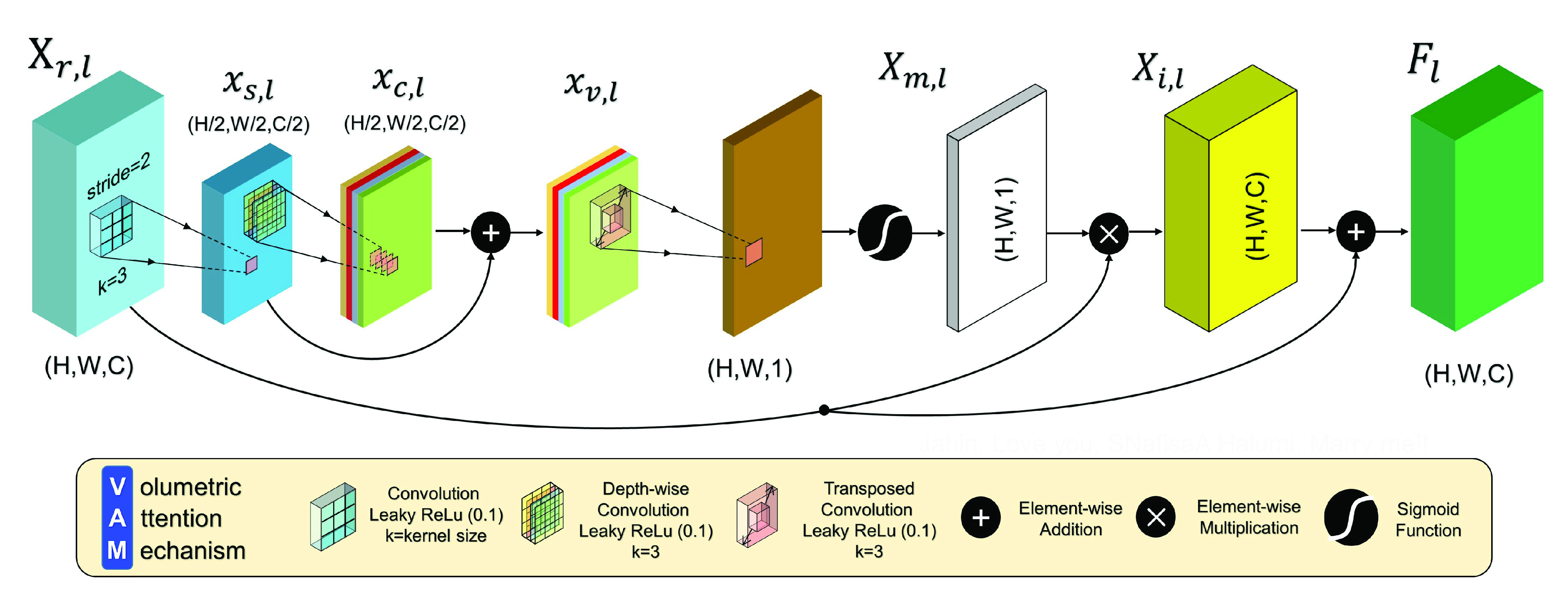
Illustration of the proposed volumetric attention mechanism (VAM). The sequential operations of strided convolution and depth-wise convolution with residual connection produces a feature map $x_{v,l}$ consisting of a mapping for higher relevant features passed on to mask the input and provide attention based on that mapping. $F_{l}$ is the highlighted version of input $X_{r,l}$.

**Fig. 5.**
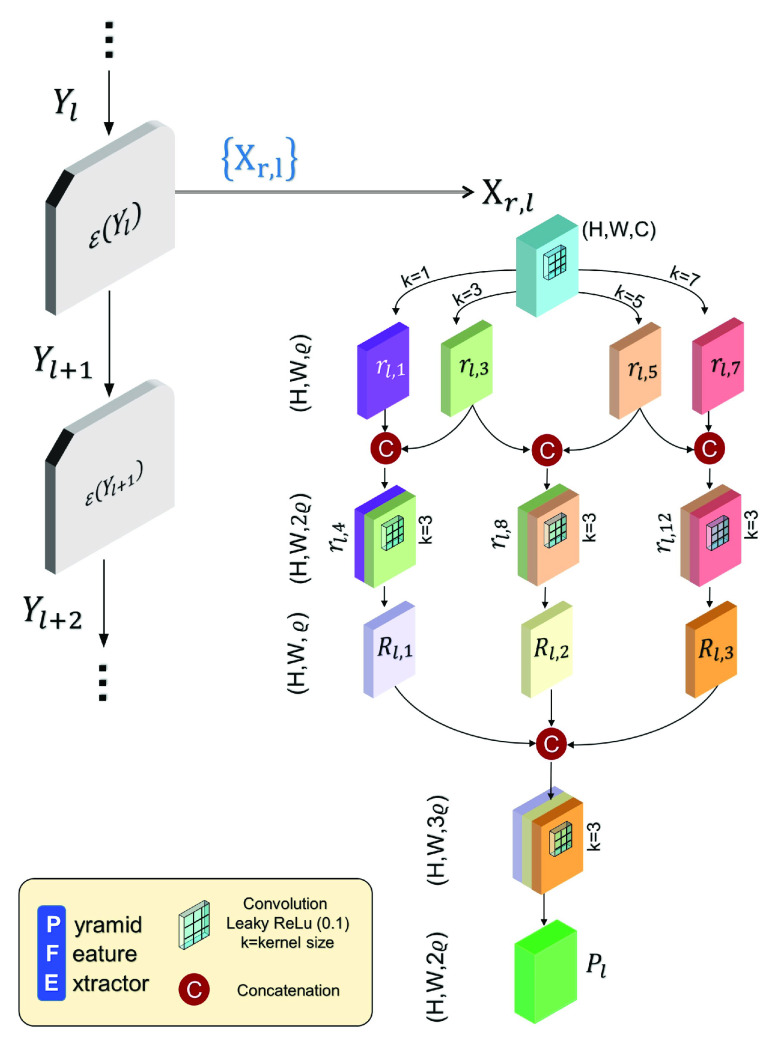
Proposed pyramid feature extractor (PFE) structure: the passed on feature map $X_{r,l}$ coming from the Compression pipeline is transformed into $P_{l}$ through PFE which contains regional quantitative information of the input.

In the proposed network, first a Compression Pipeline is designed which not only reduces the non-essential feature information, but also offer attention towards the important and relevant features. This contains a repeating Encoder Module with a volumetric attention mechanism (VAM) to handle the first challenge. In order to address the second challenge of extracting the contextual information, a Regional Correlative Pipeline is designed. It contains a transformation block known as a Pyramid Feature Extractor (PFE) to determine the regional dependence amongst features adapting with dimensional changes. Finally, after feature aggregation and propagation is complete from both pipelines alongside proper dimensional reductions, all of the features are to be examined for classifying the input data. Evidently the features coming from each of the pipelines are different. As a result, a few non-linear weighted sums of the features are taken into account to get the decision on the pathological aberrations which are likely to be present on the image. This ending pipeline is known as the Decision Pipeline. The proposed network consisting of these three pipelines is presented in Fig. 2. In the figure the aforementioned pipelines are clearly marked using three different colours. The detailed explanation of each pipeline is presented:

1) *The Compression Pipeline:* This pipeline performs volumetric compression of image driven feature maps. Initially, it takes an RGB image $I\,\,\in \,\,\text{R}^{H \times W \times 3}$ as an input and passes that through the Encoder Module. This proposed module is responsible for the feature maps’ dimension reduction as well as relevant feature accumulation and prominence preservation. The generalized version of it is depicted in Fig. 3. This encoder module takes a feature map or input $X\,\,\in \,\,\text{R}^{H \times W\times C0}$ and implements upon it a consecutive of two convolution filters to produce $X_{r}\,\,\in \,\,\text{R}^{H \times W \times C}$. The required relationships can be written as:
\begin{align*} C=&\left \{{n\left |{C_{0}=3; 2 C_{0}}\right | C_{0} \neq 3}\right \} \tag{1}\\ X_{r}=&\mathbb {W}_{2, l}^{3,1}\left ({\mathbb {W}_{1, l}^{3,1}(X)}\right)\tag{2}\end{align*}
  Here, $\mathbb {W}_{t,l}^{k,s}$ indicates a non-linear transformation which consists of $t$-th occurrence of a (*k,k*) shaped convolution filter followed by a LeakyRelu activation (*out*: *out* = *in* $\vert $ *in* $\ge0$; *out* $= \alpha \ast $ *in* $\vert $ *in* < 0 & $\alpha =0.1$) function corresponding to the $l$-th occurance of an Encoder Module. Furthermore, the filter $\mathbb {W}_{t,l}^{k,s}$ convolves through any feature tensor with $s$-strides. Here, $n$ was set to 32 for experimentation. After the convolutional layer, the norm is to introduce a Pooling layer. However, in the proposed scheme, unlike conventional approaches, prior to utilizing a pooling layer an attention mechanism is introduced in order to create high variance amongst the feature maps. The attention mechanism operation produces $F\,\,\in \,\,\text{R}^{H \times W \times C}$ which is followed by a maxpooling layer to output $Y\,\,\in \,\,\text{R}^{H/2 \times W/2\times C}$. The described Encoder Module is represented as $\varepsilon (X)$. This operation may be interpreted as mentioned below. In this case, $\mu _{k\times k}(\cdot)$ represents Max-pooling operation with a (*k,k*) shaped window:
\begin{equation*} Y = {\it \mu }_{2\times 2}(F) = {\it \mu }_{2\times 2}({\textit{Att}}(X_{r}))\tag{3}\end{equation*}
  The proposed Volumetric Attention Mechanism (VAM), which resides inside the Encoder module, has the structure depicted in Fig. 4. It creates values which would provide attention within the input in two different yet sequential methods. Initially, VAM takes an input tensor $X_{r,l} \,\,\in \,\,\text{R}^{H \times W \times C}$ which goes through a strided convolution operation with reduced filters. This outputs a feature vector $x_{s,l} \,\,\in \,\,\text{R}^{H/2 \times W/ 2\times C/2}$ containing only potent spatial information and owing to having fewer channels, it possesses relatively higher significant feature attributes only. Nevertheless, the feature attributes distributed channel-wise are not equally important with respect to each other. Therefore, a depth wise convolution is performed over the aforementioned tensor to not only differentiate the channel-wise feature attributes but also enhance the features with regards to relevance. This provides a feature map $x_{c,l}\,\,\in \,\,\text{R}^{H/2\times W/2 \times C/2}$. Next in order to reinforce the features, both spatially and depth-wise, both those features are added together. As a result, a volumetric information mapping $x_{v,l} \,\,\in \,\,\text{R}^{H/2 \times W/ 2\times C/2}$ is obtained which will be used for generating attention towards the input $X_{r,l}$ further on. The operations may be written like so:
\begin{align*} x_{c, l}=&\mathbb {D}_{l}^{3,1}\left ({x_{s, l}}\right)=\mathbb {D}_{l}^{3,1}\left ({\mathbb {W}_{3, l}^{3,1}\left ({X_{r, l}}\right)}\right) \tag{4}\\ x_{v, l}=&x_{s, l} \oplus x_{c, l}\tag{5}\end{align*}
  As these expressions shown here, $\mathbb {D}_{l}^{k,s}$ implies a non-linear transformation consisting of a (*k,k*) shaped $s$-strided depthwise convolution filter followed by a LeakyRelu activation ( $\alpha =0.1$) function within the $l$-th occurance of an Encoder Module. Moreover, $\oplus $ is used to represent element-wise summation. The volumetric mapping is afterwards convolved in a transposed manner which includes a spatial upsampling with an addition of convolution filtering. With appropriate filter counts the volumetric mapping is brought up to the original shape of the input and then the obtained feature map is passed through a sigmoid activation function. This means that a mapping feature vector is found to be $X_{m,l} ~\in $ [0, 1] $^{H \times W \times 1}$. Owing to the fact that the values residing within $X_{m,l}$ are confined between a certain range, this feature vector is effectively a mask which will attenuate the less important features while hardly tampering the significant ones. Hence, it is multiplied with the input to obtain a masked version of $X_{r,l}$, which is $X_{i,l} \,\,\in \,\,\text{R}^{H \times W \times C}$ and thereafter, added to the input to enhance as well as highlight the input feature tensor’s relevant attributes to produce the final output $F_{l} \,\,\in \,\,\text{R}^{H \times W \times C}$. The remaining operations of the attention mechanism are presented:
\begin{align*} X_{m, l}=&\sigma \left ({\mathbb {T}_{l}^{3,2}\left ({x_{v, l}}\right)}\right) \tag{6}\\ F_{l}=&X_{r, l} \oplus X_{i, l}=X_{r, l} \oplus \left ({X_{r, l} \otimes X_{m, l}}\right)\tag{7}\end{align*}
  Here, $\mathbb {T}_{l}^{k,s}$ is indicates a non-linear transformation consisting of a (*k,k*) shaped $s$-strided transposed convolution operation followed by a LeakyRelu activation ( $\alpha =0.1$) function within the $l$-th occurance of an Encoder Module. And, $\sigma $ is used for sigmoid activation; mathematically defined as $\sigma \left ({z }\right)=1/(1+e^{-z})$. In this way through the attention mechanism, any given feature tensor is optimized to output a vector having further relevant pixel values based on the training procedure.
  And since it is utilized inside the Encoder module, it plays a potent role in producing a decent extent of quantitative gap among low-grade and high-grade features prior to being Maxpooled, after which only the significant values are passed through during propagation. After numerous stages of implementing the Encoder module, the $I\,\,\in \,\,\text{R}^{H \times W \times 3}$ image turns into a tensor namely $Y_{5}\,\,\in \,\,\text{R}^{H/ 32 \times W/ 32\times 16 C}$ on which Global-Average pooling is performed afterwards to obtain a one dimensional vector $\tilde {y_{5}}\,\,\in \,\,\text{R}^{1\times 1\times 16 C}$. This vector represents the accumulated information arriving from the entire Compression Pipeline and is sent to the Decision Pipeline.
2) *The Regional Correlative Pipeline:* As mentioned before, to gather the contextual information from the image, the input to the Regional Correlative Pipeline is taken from the layer prior to employing the attention mechanism. This pipeline requires feed-forward features from the Compression Pipeline from varying dimensions as well as the parameter $N$. The structure of it includes the numerous utilizations of PFE block which is solely responsible in correlating the local region based attributes of pixels in an image space and the architecture of it is presented in Fig. 5. Initially, a registered feature tensor $X_{r} \,\,\in \,\,\text{R}^{H\times W \times C}$ produced by the $l$-th occurrence of Encoder Module is taken into account and convolved over parallelly using different kernel shapes with $\varrho $ number of channels. The resulting features maps are paired-wise concatenated to estimate their mutual relation. This operation is expressed as:
\begin{align*} r_{l, 2j+1}=&\mathbb {W}_{4+j, l}^{2 j+1,1}\left ({X_{r, l}}\right);\quad \forall j \in \{0,1,2,3\} \tag{8}\\ r_{l, 4j+4}=&{Con}\left [{r_{l, 2j+1}, r_{l, 2j+3}}\right];\quad \forall j \in \{0,1,2\}\tag{9}\end{align*}
  Here, *Con* $[.]$ means the concatenation of feature maps along the final axis. Each $r_{l, 4j+4}\in \,\,\text{R}^{H\times W \times 2\varrho }$ is then convolved, fused, and finally concatenated to gather each of the correlative attributes together. Finally, one more non-linear convolution is performed to fuse the resulting feature map into a singular feature vector $P_{l}\,\,\in \,\,\text{R}^{H \times W \times 2\varrho }$. All these implementations can be expressed as:
\begin{align*} R_{l, j}=&\mathbb {W}_{3, l}^{7+j, 1}\left ({r_{l, 4j}}\right); \quad \forall j \in \{1,2,3\} \tag{10}\\ P_{l}=&\mathbb {W}_{3, l}^{11,1}\left ({{Con}\left [{R_{l, 1}, R_{l, 2}, R_{l, 3}}\right]}\right)\tag{11}\end{align*}
  As a result, a tensor $P_{l} $ produced from the $l$-th occurrence of an Encoder module possesses the cumulative characteristics describing the effective regional correlation which in sense would address the surrounding mucosal view along with the abnormality itself constructively. In the end of the Regional Correlative Pipeline, each $P_{l}$ is faced with Global-Average pooling to determine the channel-wise mean values which are represented by $\tilde {p_{l}}\,\,\in \,\,\text{R}^{1\times 1\times 2\varrho }$; $\forall l~ \in $ {2,3,4,5} and the outputs are passed onto the Decision Pipeline for the final stage of the classification procedure.
3) *Decision Pipeline:* The 1D feature maps which generate from the previous pipelines are concatenated. This pipeline uses fully connected dense layers to aggregate 1D features arriving from the previous pipelines and multiplies optimizable weights to them with a view to perform weighted summing. As such, relevant features even among the 1D vectors are enhanced to make the final classification easier. The structure of the Decision pipeline is shown in Fig. 2. After concatenation, two LeakyRelu activation ( $\alpha =0.1$) functions with two dense layers (512, 128) are connected. In this way the features are being condensed into smaller dimensions. Finally, a softmax or sigmoid activation is added based on how many classes of are being considered and a final decision is formulated.

## V. Result and Analysis

### A. Implementation Detail

In order to build and train the model along with all the necessary data management steps, cloud GPU provided by Google Colaboratory is utilized with Keras library. The data distribution in each class of diseases is shown in Table 1. It is observed that the dataset containes a very large number of WCE images from normal class in comparison to all other diseased classes (about more than 40 times). During dealing with binary class or multi-class problem, the smallest class is first randomly split into 80%-20%. The value yielded by that 20% is considered as a reference value for each of the other classes. replicated for each of the other classes and each 20% was included in the validation set. Next each of the 80% data are upsampled using the mentioned augmentation techniques to obtain around 3000 images per class. As a result, the training set is also created. Along with our proposed model, two additional models mentioned in [13], namely AlexNet [14] and GoogLeNet [15], are also used for comparison purposes.

### B. Evaluation Matrices

Precision (Prec), Recall (Rec), F1-score and Accuracy (Acc) are standard matrices. These matrices, used for the purpose of evaluation, are expressed as:

\begin{align*} {Prec}=&\frac {\sum T P}{\sum (T P+F P)} \tag{12}\\ {Rec}=&\frac {\sum T P}{\sum (T P+F N)} \tag{13}\\ F 1-s c o r e=&2 * \frac {Prec * Rec}{Prec+Rec} \tag{14}\\ A c c=&\frac {\sum (T P+T N)}{\sum (T P+T N+F P+F N)}\tag{15}\end{align*}

Here, TP, TN, FP, FN are defined as True-Positive, True-Negative, False-Positive and False-Negative respectively.

### C. Result and Analysis

In binary classification, the objective is to classify the normal and the GI diseased class. In that case, 5 different cases were considered, such as normal Vs. ulcer, normal Vs. erosion and normal Vs. bleeding, normal Vs. lymphangiectasia and normal Vs. angiectasia. Each of these binary classifications are evaluated with matrices mentioned before and the mean values are shown in Table 2 (N = Normal, U = Ulcer, E = Erosion, B = Blood, S = Single Abnormality). The ‘N-S’ based performance is shown as the average of all normal and abnormal 2-class cases. Next, a 3 class and a 4 class training were also taken into account. Performance of the proposed scheme for binary, 3 class and 4 class problems are shown in Table 2; whereas the two widely used CNN methods namely AlexNet and GoogLeNet are taken for comparison. Here, these two CNN networks are taken along with the proposed RAtCapsNet and just as the proposed one, they are not pre-trained with any external data.

**TABLE 2:** Comparison Among Classification Performance of Different Number of Classes

Class	Network	Prec	Rec	F1	Acc
N-U-E	RAt-CapsNet	0.9792	0.9792	0.9792	0.9792
	AlexNet	0.9353	0.9353	0.9353	0.9353
	Google-Net	0.9545	0.9526	0.9535	0.9526
N-U-B	RAt-CapsNet	0.9883	0.9883	0.9883	0.9883
	AlexNet	0.9805	0.9766	0.9785	0.9766
	Google-Net	0.9766	0.9766	0.9766	0.9766
N-U-E-B	RAt-CapsNet	0.9563	0.9538	0.9550	0.9565
	AlexNet	0.9373	0.9348	0.9360	0.9348
	Google-Net	0.9399	0.9375	0.9386	0.9402
N-S	RAt-CapsNet	0.9837	0.9873	0.9858	0.9851
	AlexNet	0.9760	0.9445	0.9594	0.9611
	Google-Net	0.9772	0.9855	0.9813	0.9812

Firstly, in Table 2 for binary classes, it is clearly evident that the proposed framework outperforms the other two networks in case of all kind of evaluation matrices. The average highest and lowest accuracy recorded was 98.51% and 96.11%, obtained from our proposed ‘RAt-CapsNet’ framework and Alexnet accordingly. In normal - ulcer and normal - erosion our proposed network has shown the highest accuracy compared to the other two networks. However, due to lack of distinguishing attributes between normal-lymphangiectasia and normal-angiectasia classes, all networks provide relatively lower accuracy. On the contrary, due to clearly distinguishable bleeding images, normal-bleeding classification offers the best performance for all networks.

In multi-class outcomes presented in Table 2, there is no exception here. The proposed framework clearly outperforms all other networks in each evaluation matrices. The improvement of true result values in the proposed scheme implies a better outcome in precision, recall, F1-score and accuracy. For example, in the normal- ulcer- erosion variation of the multiclass, RAt-CapsNet achieves an accuracy of 97.92%, whereas AlexNet and GoogleNet fail to obtain better accuracy than 93.53% and 95.26% accordingly.

In terms of total parameters, the computational complexity of RAt-CapsNet, it is found to have a total of 9.56 million parameters whereas AlexNet has a total parameter of 37.34 million and GoogleNet has a total parameter of 6.99 million. Considering very satisfactory classification performance, such a computation complexity of the proposed framework is quite acceptable.

**Fig. 6.**
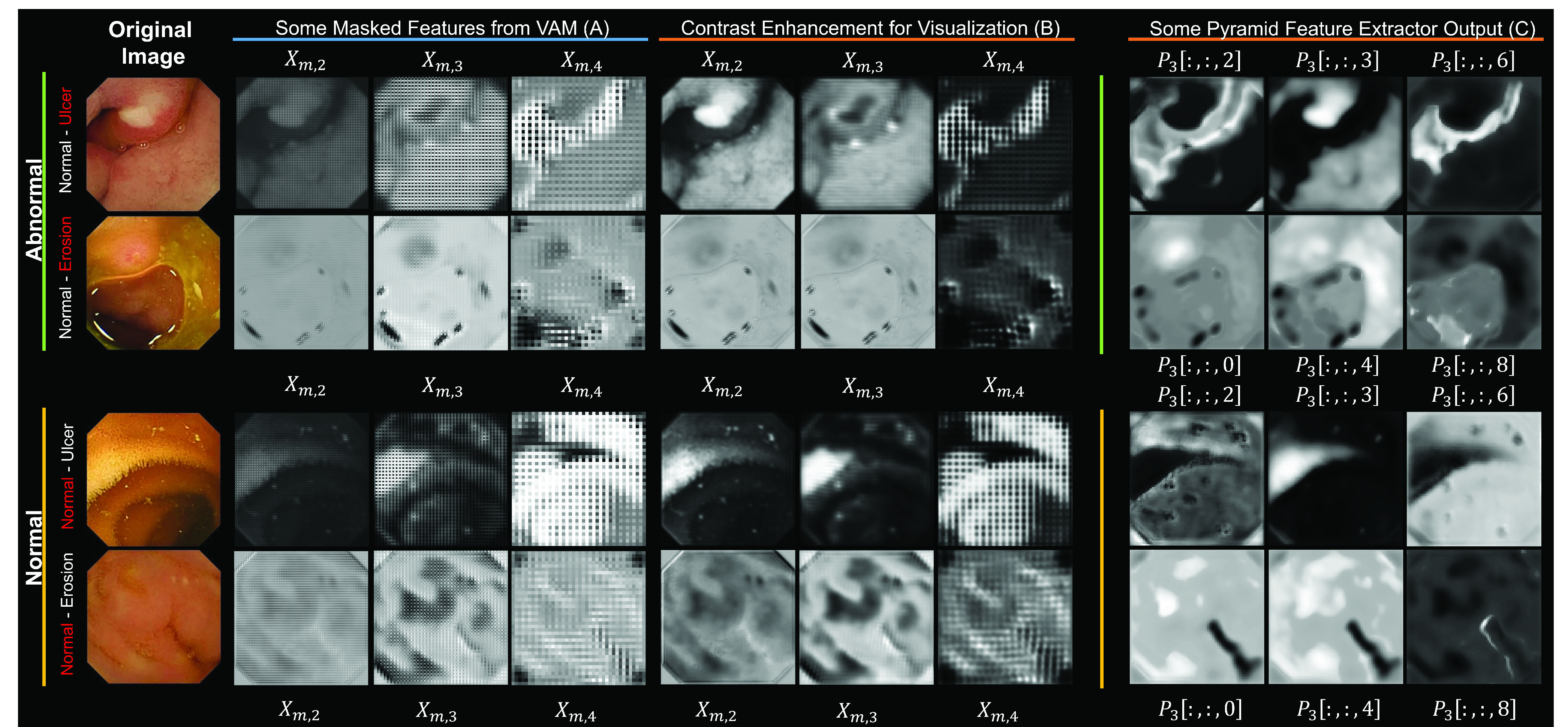
Instances of various dimensional masks generated by the volumetric attention mechanism as well as some output from a Pyramid Feature Extractor block; all of which produced and gathered from some exemplary abnormal and normal images. Columns marked ‘B’ are the contrast stretched version of columns marked ‘A’ produced from masked version of the 2nd attention mechanism. Columns marked ‘C’ contain three feature maps from $P_{3}$, output of PFE.

In order to present the proper effectiveness of the proposed VAM and PFE blocks, 4 sampled cases are considered in Fig. 6. The first usages of the attention ( $X_{m,2}$, $X_{m,3}$) create a noticeable boundary across the abnormality which captures the abnormal representing features. After further usage ( $X_{m,4}$) it creates tarnished masks for the abnormal images (ulcer and erosion from the 2 class training are shown) and creates high average intensity variance among the classes. The images of the first and third rows from Fig. 6 arrive from the ‘Normal-Ulcer’ trained model. Similar argument goes for the second and fourth row based on ‘Normal-Erosion’. Columns marked ‘A’ contain masked images which have been contrast-enhanced for visualization and put in columns marked ‘B’.

Additionally, the PFE block tries to find the tentative locale of the abnormality shown in columns marked ‘C’. Three feature maps among $2\varrho $ residing within $P_{3}$ are shown as examples. The anomaly-based probable, yet limited, areas get marked distinctly with either high or low values. For this, the Regional-Correlative Pipeline produces alternate values (channel-wise) after global average pooling for normal and abnormal images. This distinct distribution of values is useful for the dense layers in the Decision pipeline.

## VI. Discussion Compared to Former Works

In the cases of Normal-vs-(Ulcer/Erosion/Blood), RAt-CapsNet provides accuracies of 0.9971, 0.9854, and 1.00, respectively. In comparison to these results, for the case of ulcer data, accuracies for normal-vs-ulcer events were reported as 0.9516 and 0.967 by [25] and [16]. For erosion, accuracy for normal-vs-erosion was reported as 0.9534 by [25]. Finally, for normal-vs-blood, [21], [22], [7] report accuracies of 0.99, 0.9989 and 0.9617, respectively. However, such comparison is not entirely fair, because the WCE datasets used by these former works were all private datasets and have imaging as well as number discrepancies with respect to Kvasir Capsule. Nevertheless, the results of the proposed RAt-CapsNet algorithm provide very satisfactory performance.

## VII. Conclusion

In this paper, a proposed dual pipelined network, RAtCapsNet, consisting of both attention and multi-dimensional regional feature extraction is adopted with a view to efficiently predicting WCE anomalies residing in the GI tract. The attention based operations make sure to generate high enough variance among healthy mucosal image and images representing pathological irregularities. Moreover, the Pyramid Feature Extractor introduces regional modification to an image based on there being abnormalities in the surrounding area. Combining both of these advantages provide highly satisfactory results in distinguishing WCE images with various attributes in both binary and multi-class scenarios. The low resolution images having discerning data structure leads to high F1-scores and Accuracies which is validated by extensive experimentation. The dataset contains significant class imbalance. However, even for the class containing less number of members (e.g. Lymphangiectasia), the proposed scheme exhibits decently satisfactory results. In broad context, RAt-CapsNet proves to be a methodical way in identifying GI abnormalities with substantial performance in wireless capsule endoscopy and has the potential to serve as a promising diagnostic tool.
